# Highlights from the 57th Bürgenstock Conference on Stereochemistry 2024

**DOI:** 10.1039/d4sc90102a

**Published:** 2024-06-13

**Authors:** Jesús Mosquera, Alessandro Bismuto

**Affiliations:** a Universidade da Coruña, CICA – Centro Interdisciplinar de Química e Bioloxía Rúa as Carballeiras 15071 A Coruña Spain j.mosquera1@udc.es; b Institute of Inorganic Chemistry, University of Bonn Gerhard-Domagk-Str. 1 53121 Bonn Germany bismuto@uni-bonn.de

## Abstract

Herein, we share an overview of the scientific highlights from speakers at the latest edition of the longstanding Bürgenstock Conference.
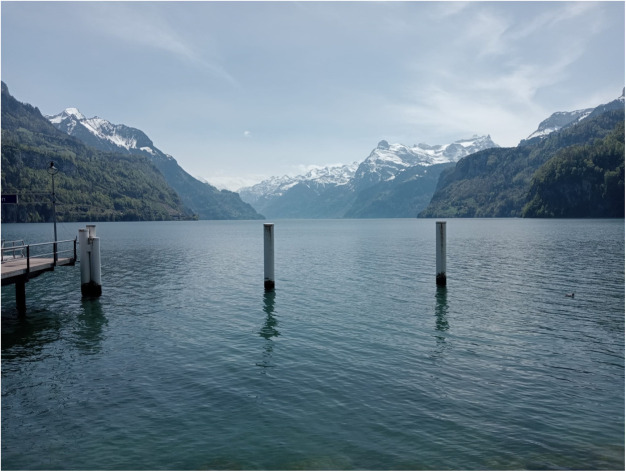

## Introduction

“I give them experiments and they respond with speeches.” – Louis Pasteur.

As the tradition goes, at the beginning of May 2024, the 57th edition of the Bürgenstock Conference was hosted in the breathtaking landscape of Brunnen, Switzerland. This exceptional venue has, for years, provided an ideal atmosphere for full-time scientific engagement over five days. Both young and established scientists gather to discuss cutting-edge challenges, extending beyond stereochemistry to encompass the entire field of chemistry. The president this year was Prof. Erick Carreira (ETH Zürich) who was assisted in welcoming all the guests by incredibly good weather. Everything started with a meet-up of the JSP fellows, with awardees coming from all over the world, including the USA and Asia, this year. This intriguing and secret event was then ready to start with the announcement of the guest of honor, Prof. Scott E. Denmark (University of Illinois, Urbana-Champaign), and a few words from this year’s vice president, Prof. J. L. Mascareñas (Universidade de Santiago de Compostela).

## The RNA world

The inaugural lecture on Sunday evening, chaired by Vice President Prof. J. L. Mascareñas, was delivered by Nobel laureate Prof. Jack Szostak from the University of Chicago on the topic of the origin of life. As a leading exponent of the RNA world hypothesis, Prof. Szostak focused his presentation on non-enzymatic template-directed RNA replication, which is proposed as a transitional phase preceding the emergence of ribozyme-catalyzed genetic information copying.^1^

In the first part of his presentation, Prof. Szostak discussed the role of imidazole derivatives as potent activating agents for nucleotide coupling, potentially enabling RNA replication in protocells.^2^ He then highlighted the current limitations of non-enzymatic copying in relation to the origin of life and proposed the virtual circular genome model as a solution to these challenges.^3^

## Preparing polymers with CO_2_

Monday commenced with a lecture delivered by Prof. Charlotte Williams (University of Oxford). She grounded us in the reality of our present world, emphasizing the significant journey ahead in achieving the essential reductions in greenhouse gas emissions required to meet global climate targets, with a specific focus on the challenges posed by plastics.^4^ Following this, she introduced her remarkable heterodinuclear catalysts, which facilitate the utilization of carbon dioxide as a feedstock to substitute virgin petrochemicals in polycarbonate synthesis. These synergistic and highly active catalysts, composed of inexpensive metals, enable the ring-opening copolymerization of epoxides and carbon dioxide.^5^ They operate through a dinuclear metalate mechanism, where one metal binds and activates monomers while the other binds the polymer, easing the insertion into the activated monomer in close proximity.^6^

## Smart molecular cages

Introducing a captivating conceptual shift, Prof. Niveen Khashab took to the stage to unveil a range of innovative applications for organic molecular cages. Initially, she showcased a small molecular cage designed to shield proteins from insolubility issues induced by the Hofmeister effect.^7^ However, the highlight of her presentation centered on the fusion of cages with organic polymers. Exploring this synergy, she elucidated its numerous applications, ranging from enhancing molecular separation techniques^8^ to enabling touchless technology.

One of the most remarkable aspects of her presentation focused on materials that demonstrate mechanical responses to vapor stimuli, swiftly returning to their original shape once the stimuli are removed. She effectively demonstrated the applicability of these materials through engaging videos, showing how objects can be gripped and released depending on the solvent vapor.^9^

## Enzymes: Nature's Swiss Army knife

After a fantastic lunch, the afternoon session started. Prof. Rebecca Buller emphasised the expansive chemical space accessible for protein design and its potential utility in developing novel enzymes through direct evolution. She then discussed some illustrative examples involving the development of an aliphatic halogenase capable of incorporating chloride anions onto unactivated sp^3^-hybridized carbon centers of pharmaceutically relevant molecules, such as the martinelline core and soraphens.^10,11^

We also heard about anthocyanins, plant pigments extensively utilized in various technological applications. These pigments cannot be synthesized outside of plants and must be extracted from raw materials. Prof. Buller clarified that this limitation arose from the misconception that the protein anthocyanin-related glutathione transferases, predominantly linked to anthocyanin transport, also play a role in the crucial dehydration of the flavan-3,3,4-triol intermediate.^12^

## Poster session

Monday and Wednesday afternoons were allocated for poster sessions. A total of thirty-eight posters, presented by researchers across different career stages, sparked lively discussions encompassing various fields of chemistry. Preceding the poster session, twelve junior participants were chosen to deliver 9-minute presentations. Interestingly, the conclusion of each presentation was politely announced by the sound of a duck this year.

## Illuminating chemical transformations

The final session of Monday was led by Prof. Corey Stephenson. His presentation began with his immediate transition from the University of Michigan to the University of British Columbia.

Following this, he moved to the chemical aspect of his presentation, exploring the multitude of discoveries made by his group in redox catalysis. Some of these breakthroughs included: (i) advancing new analogues of the phthalimide *N*-oxyl (PINO) with higher stability to catalyze hydrogen atom transfer reactions,^13^ (ii) employing a photoredox-mediated N-centered radical strategy to facilitate carboamination/dearomatization cascade reactions,^14^ and (iii) using a photoredox reaction to synthesize 2,2-diarylethylamines from arylsulfonylacetamides.^15^ In the final segment of the presentation, Prof. Stephenson delved into the design of photoreactors for scaling up visible-light photochemical reactions in flow, utilizing simple equipment.

## Functionalizing sp^3^ carbons

Prof. Mariola Tortosa (Universidad Autónoma de Madrid), a former JSP fellow on the 48th Bürgenstock Conference in 2013, inaugurated the Tuesday session. Prof. Tortosa's presentation highlighted the medicinal chemistry community's strategic shift towards leveraging sp^3^ carbons to enhance clinical efficacy. In response, her team developed borylation methodologies targeting sp^3^ carbons.^16^ Noteworthy achievements included the enantioselective synthesis of cyclobutylboronates and a diastereo- and enantioselective base-promoted diboration process for spirocyclobutenes.^17^ In the culmination of her discourse, Prof. Tortosa elucidated synthetic pathways harnessing amino moieties to introduce novel functionalities, exemplified by the substitution reaction of propargylic ammonium salts with aryl Grignard reagents.^18^

## Dealing with flexibility

Prof. Sereina Riniker (ETH Zürich) was the next speaker, sharing how her group has built a research program around dealing with the conformational flexibility of biomolecules by using molecular dynamics. Firstly, Dr Riniker explored the intricacies of the folding and stability of biomimetic synthetic analogues of collagen.^19^ Secondly, she showcased computational models enabling the prediction of gas-phase peptide conformations. Subsequently, she elucidated the mechanisms governing the permeation of cell membranes by cyclic peptides.^20^ Concluding her presentation, she introduced an innovative model utilizing vibrational circular dichroism (VCD) for enantiomer identification, offering promising applications in the field.^21^

## Going beyond alkene metathesis

After a leisurely Tuesday afternoon where participants enjoyed the sunny weather and the natural landscapes of Brunnen, the scientific sessions resumed post-dinner with a presentation by Prof. Corinna Schindler (University of Michigan). However, before her talk, the organizing committee allocated a few minutes to discuss Asian conferences with parallels to the Bürgenstock, specifically highlighting the Tateshina and Bowei research conferences.

Prof. Schindler started by disclosing her renowned research on carbonyl–olefin metathesis catalyzed by iron(iii).^22^ Initially confined to aryl ketones, her group successfully broadened the scope to encompass aliphatic ketones. Subsequently, she introduced an aluminum-based heterobimetallic ion pair catalyst, demonstrating superior performance and facilitating carbonyl–olefin ring-closing metathesis to access six- and seven-membered rings.^23^ The second topic was the development of visible-light-mediated intermolecular [2 + 2] photocycloadditions for the synthesis of azetidines.^24^ Finally, she inspired everyone by presenting how to apply this last reaction to perform RNA labelling.

## RoboChem, pushing the boundaries of synthetic chemistry

The morning session started with an innovative presentation from Prof. Timothy Noel (University of Amsterdam) on how much technology can not only influence our lives, but also the usual paradigm of performing chemistry. The first part focused on an important topic in synthesis: can we find a safe way to generate thionyl fluorides and use it to functionalise molecules?^25^ The answer is yes, and flow is key to doing it in a safe way. Flow chemistry was additionally exploited to promote arylation of alkanes using light and a nickel catalyst.^26^ Furthermore, he revealed the story and the excitement around RoboChem – an exciting approach to access fast optimization in catalysis.^27^ This platform used a combination of software and hardware to control and automatize photocatalytic reactions in flow; the final icing on the cake was the combination with an in-line NMR to collect the data and improve the optimization. Will that change the view of performing reaction optimization?

## Voice to main-group elements

After the coffee break, it was time to switch topics, moving towards main-group chemistry with Prof. Manuel Alcarazo (University of Göttingen). The presentation was a real roller coaster pushing the boundaries of main-group chemistry, starting from electron-deficient phosphine ligands to non-innocent phosphorous complexes and sulfur reagents. Exquisite ligand design led to the isolation of reactive square pyramidal phosphorous compounds and through P-ligand cooperativity allowed the development of a catalytic process for the disproportionation of 1,2-diphenylhydrazine.^28^ The design moved towards reagents, with sulfonium species not only for C–C coupling,^29^ but now entering the field of skeletal editing. Starting from benzothiophene, he and his team were able to synthesize a new nitrene reagent, on a gram scale, that can be used in combination with a rhodium catalyst to generate isoquinolines from indenes.^30^

## Digital chemistry in drug discovery

The afternoon session started with Dr Antonia Stepan (Roche), focusing on the recurring topic in this meeting: the effect of technology in medicinal chemistry and late-stage functionalization. Specifically, the use of new technology and high-throughput experimentation can not only help in the purification steps but also assist in successfully identifying reaction conditions. In addition, this has lately been further extended to reaction prediction where computational methods can be used to predict reaction yields with good accuracy.^31^ The prediction can be further extended to solubility and other intermolecular properties of a specific target; for instance, increasing the lipophilicity of a compound can help in entering the cavity of a protein or help the assimilation by the body. The synthetic achievement of this is combined with artificial intelligence (AI), carrying out virtual screenings to identify promising candidate hits. Finally, the anticipation of drug properties, mainly focusing on half-life and dosage prediction, was also presented, leveraging software to forecast *in vivo* behavior during drug development.

## Understanding and prediction in transition-metal catalysis

This intense day culminated with the last speaker of the day, Prof. Franziska Schönebeck (RWTH Aachen University) whose talk was set at the boarder between physical organic chemistry and catalysis. Through the use of computational chemistry, data analysis and mechanistic investigation, she and her group were able to shed light on major challenges in catalysis. The first topic touched on was Pd(i) dinuclear and mononuclear catalysis, with the initial discussion centering on an unexpected and air-sensitive complex that was stabilized *via* ligand design. A highlight of the application of this compound in catalysis was the isomerization of olefins and the selective formation of vinyl cyclopropanes.^32^ Within the same group, the presentation proceeded with the use of machine learning to predict stable nickel(i) complexes and their application in C–C cross-coupling.^33^ As final diversification, Prof. Schönebeck moved towards main-group elements, highlighting the potential of germanium compounds in organic synthesis.^34^

## Challenges and opportunities in natural product synthesis

Prof. Thomas Magauer (University of Innsbruck), a JSP fellow in 2015, opened up the last morning session by going back to the origin of this meeting, stereochemistry. His talk was a marvelous trip through the art of disconnection in total synthesis. Diterpenoids are important targets in total synthesis which present several stereocenters whose installation requires *ad hoc* protocols and a perfect sequence of addition and cyclisation.^35^ The presentation continued with discussion on the total synthesis of a sesquiterpenoid, which was achieved in less than 10 steps featuring *N*-terminated cyclisation.^36^ In the last session, Professor Magauer delved into unpublished work related to the synthesis of meroterpenoids.

## From shuttle catalysis to molecular editing

The meeting concluded with fireworks. Prof. Bill Morandi (ETH Zürich) was the last on stage keeping the attention and interest focused with a saga that went from functional-group transfer to single-atom editing. The talk started with the famous concept of shuttle catalysis, followed by the origin of nickel-catalyzed CN transfer to C–S and C–P metathesis, and continued with mechanistic investigations to improve the catalyst performance and the application in materials.^37^ The icing on the cake was the extension of this concept to an e-shuttle for dehalogenation reactions. The line of research further extends to fundamental organometallic chemistry, and how we control and predict single steps in organometallic chemistry, such as β-hydride elimination.^38^ Last but not least is skeletal editing, where using electrophilic reagents together with his team he was able to selectively add a nitrogen atom to industrial and biologically relevant compounds, generating a new approach at a molecular level.^39^

## Final remarks

After all these unique and inspiring talks, the meeting closed with general remarks from the current president, followed up with significant highlights from the organizing committee. Of particular note was the great participation this year, both in terms of general attendance from all over the globe to the Q/A sessions that always had a long list of stimulating questions. Automation, robots, and artificial intelligence are becoming more and more relevant to organic synthesis, catalysis and chemistry in general; this topic was central to many debates – what will a laboratory look like in ten years?

## Author contributions

Both authors contributed to the drafting of this conference report.
